# Bis(2-amino-6-methyl­pyridinium) *trans*-diaqua­bis­(pyrazine-2,3-dicarboxyl­ato)cuprate(II) hexa­hydrate

**DOI:** 10.1107/S1600536811008981

**Published:** 2011-03-15

**Authors:** Hossein Eshtiagh-Hosseini, Azam Hassanpoor, Masoud Mirzaei, Teresa Szymańska-Buzar, Andrzej Kochel

**Affiliations:** a Department of Chemistry, School of Sciences, Ferdowsi University of Mashhad, Mashhad, Iran; b Faculty of Chemistry, University of Wrocław, F. Joliot-Curie 14, 50-383 Wrocław, Poland

## Abstract

The title compound, (C_6_H_9_N_2_)_2_[Cu(C_6_H_2_N_2_O_4_)_2_(H_2_O)_2_]·6H_2_O, was obtained by the reaction of CuCl_2_·2H_2_O with pyrazine-2,3-dicarb­oxy­lic acid (pyzdcH_2_) and 2-amino-6-methyl­pyridine (2a-6mpy) in aqueous solution. The Cu^II^ atom is located on an inversion centre and has an overall octa­hedral coordination environment. Two N and two O atoms from (pyzdc)^2−^ ligands define the equatorial plane and two water mol­ecules are in axial positions, resulting in a typical tetra­gonally Jahn–Teller-distorted environment. Extensive classical O—H⋯O, O—H⋯N and N—H⋯O and non-classical C—H⋯O hydrogen bonds, as well as π–π stacking inter­actions between aromatic rings of the cations [centroid–centroid distance = 3.58 (9) Å], lead to the formation of a three-dimensional supra­molecular structure.

## Related literature

For background to this class of compounds, see: Aghabozorg *et al.* (2008 ▶, 2010 ▶). For related structures, see: Eshtiagh-Hosseini *et al.* (2010*a* ▶,*b* ▶,*c* ▶, 2011 ▶); Che *et al.* (2009 ▶).
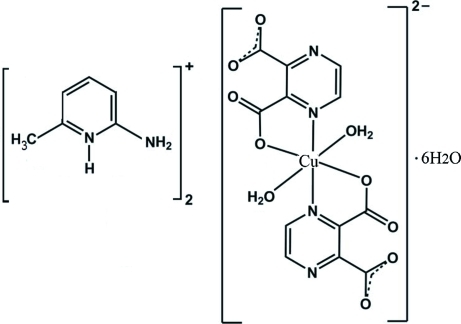


## Experimental

### 

#### Crystal data

(C_6_H_9_N_2_)_2_[Cu(C_6_H_2_N_2_O_4_)_2_(H_2_O)_2_]·6H_2_O
*M* *_r_* = 758.16
Triclinic, 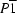

*a* = 6.7353 (3) Å
*b* = 8.0757 (4) Å
*c* = 15.0170 (6) Å
α = 79.450 (4)°
β = 86.320 (4)°
γ = 89.828 (4)°
*V* = 801.31 (6) Å^3^
*Z* = 1
Mo *K*α radiation
μ = 0.77 mm^−1^
*T* = 100 K
0.20 × 0.18 × 0.18 mm

#### Data collection

Oxford Diffraction KM-4-CCD diffractometer
Absorption correction: analytical (*CrysAlis RED*; Oxford Diffraction, 2010) ▶ *T*_min_ = 0.845, *T*_max_ = 0.910
7090 measured reflections
3758 independent reflections
3230 reflections with *I* > 2σ(*I*)
*R*_int_ = 0.015

#### Refinement

*R*[*F* ^2^ > 2σ(*F* ^2^)] = 0.030
*wR*(*F* ^2^) = 0.082
*S* = 1.09
3758 reflections
224 parameters
H-atom parameters constrained
Δρ_max_ = 0.55 e Å^−3^
Δρ_min_ = −0.21 e Å^−3^

### 

Data collection: *CrysAlis CCD* (Oxford Diffraction, 2010) ▶; cell refinement: *CrysAlis RED* (Oxford Diffraction, 2010) ▶; data reduction: *CrysAlis RED* ▶; program(s) used to solve structure: *SHELXS97* (Sheldrick, 2008 ▶); program(s) used to refine structure: *SHELXL97* (Sheldrick, 2008 ▶); molecular graphics: *DIAMOND* (Brandenburg & Putz, 2005 ▶); software used to prepare material for publication: *SHELXL97*.

## Supplementary Material

Crystal structure: contains datablocks global, I. DOI: 10.1107/S1600536811008981/wm2462sup1.cif

Structure factors: contains datablocks I. DOI: 10.1107/S1600536811008981/wm2462Isup2.hkl

Additional supplementary materials:  crystallographic information; 3D view; checkCIF report

## Figures and Tables

**Table 1:** Selected bond lengths (Å)

Cu1—O1	1.9522 (10)
Cu1—N1	1.9882 (13)
Cu1—O1*W*	2.4484 (13)

**Table 2:** Hydrogen-bond geometry (Å, °)

*D*—H⋯*A*	*D*—H	H⋯*A*	*D*⋯*A*	*D*—H⋯*A*
O1*W*—H1*W*⋯O5*W*^i^	0.83	1.97	2.7841 (17)	166
O1*W*—H2*W*⋯O6*W*^ii^	0.85	2.18	3.0199 (17)	172
O5*W*—H5*W*⋯O6*W*	0.82	2.05	2.8640 (17)	173
O5*W*—H6*W*⋯O7*W*^iii^	0.82	1.96	2.7839 (16)	175
O6*W*—H7*W*⋯N2	0.80	2.19	2.9688 (17)	162
O6*W*—H8*W*⋯O4^iv^	0.82	1.99	2.7921 (16)	168
O7*W*—H9*W*⋯O2^iv^	0.78	1.97	2.7559 (15)	177
O7*W*—H10*W*⋯O3	0.86	1.87	2.7221 (16)	176
N11—H11⋯O4	0.80	1.95	2.7522 (16)	175
N12—H12*B*⋯O3	0.80	2.06	2.8623 (17)	172
N12—H12*C*⋯O7*W*^v^	0.85	2.05	2.9014 (17)	178
C5—H5⋯O1*W*^iv^	0.95	2.53	3.3206 (19)	141
C6—H6⋯O5*W*^ii^	0.95	2.38	3.2485 (19)	151
C13—H13⋯O2^vi^	0.95	2.53	3.4081 (19)	153
C16—H16*B*⋯O2^iii^	0.98	2.58	3.2419 (19)	125

Symmetry codes: (i) 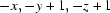; (ii) 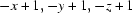; (iii) 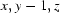; (iv) 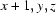; (v) 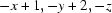; (vi) 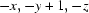.

**Fig. 1.**
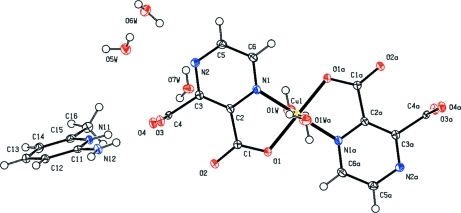
The molecular structure of the title molecule, with the atom-numbering scheme. Displacement ellipsoids are drawn at the 50% probability level. [Symmetry code a): -x, -y+2, -z+1.]

**Fig. 2.**
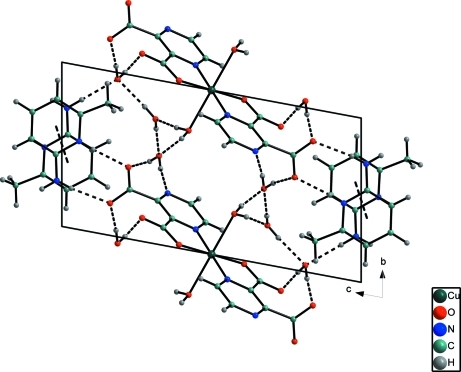
The packing diagram of the title compound, showing the supramolecular structure. The intermolecular C—H···O, N—H···O, O—H···O, and O—H···N hydrogen bonds are shown as dashed lines.

## supplementary crystallographic information

### Comment

In recent years, supramolecular complexes have attracted extensive attention owing to their potential applications. In this context, our research group has made several attempts to prepare supramolecular crystalline coordination compounds based on proton–transfer mechanism between dicarboxylic acids and amines (Eshtiagh-Hosseini, *et al.*, 2010*a*, 2010*b*, 2010*c*, 2011). Proton transfer mechanisms play a basic role in construction of supramolecular coordination compounds and water clusters (Aghabozorg *et al.*, 2008, 2010). In particular, pyrazine-2,3-dicarboxylic acid provides different modes of coordination to the metal ions (Che *et al.*, 2009). Therefore, the anion of this acid is well-know to act as a suitable ligand, especially in the design and construction of supramolecular networks. Herein, we describe the molecular and supramolecular crystal structure of a new compound, 1, with chemical formula (2a-6mpyH)_2_[Cu(pyzdc)_2_(H_2_O)_2_].6H_2_O, where pyzdcH_2_= pyrazine-2,3-dicarboxylic acid and 2a-6mpy = 2-amino-6-methylpyridine.

Fig. 1 shows the coordination environment of the Cu^II^ ion (site symmetry 1). The coordination sphere can be described as distorted octahedral, with two N and two O atoms from (pyzdc)^2-^ ligands defining the equatorial plane and two water molecules in axial positions. The Jahn-Teller effect, as observed for numerous Cu^II^ complexes, results in the elongation of the two axial Cu—O bonds towards a strong tetragonal distortion. The molecular entities of 1 consist of a [Cu(pyzdc)_2_(H_2_O)_2_]^2-^ anion, a (2a-6mpyH)^+^ cation and uncoordinated water molecules in a 1:2:6 molar ratio.

For the three–dimensional supramolecular structural set-up, extensive *X*—H···O (*X* = O, N, and C) and O—H···N  hydrogen bonding interactions as well as π—π stacking interactions between aromatic rings of the cations with a centroid—centroid distance of 3.589 Å are responsible (Fig. 2).

### Experimental

A solution of pyzdcH_2_ (0.6 mmol, 0.1 g) and 2a-6mpy (1.2 mmol, 0.13 g) in water (10 ml) was refluxed for 1 h, then a solution of CuCl_2_^.^2H_2_O (0.2 mmol, 0.01 g) was added dropwise and refluxing was continued for 6 h at 343 K. The obtained blue solution yielded blue block-like crystals of the title compound after slow evaporation of the solvent at room temperature.

### Refinement

The H atoms were generated geometrically and refined using a riding model, with C—H = 0.95–0.98 Å and *U*_iso_(H) = 1.2, 1.5 *U*_eq_(C). H atoms bonded to water molecules and nitrogen atoms were found from difference maps and than fixed. They were finally refined in the riding model approximation with riding model, with *U*_iso_(H) = 1.2*U*_eq_(N) and 1.5*U*_eq_(O).

### Crystal data

**Table:**

(C_6_H_9_N_2_)_2_[Cu(C_6_H_2_N_2_O_4_)_2_(H_2_O)_2_]·6H_2_O	*Z* = 1
*M**_r_* = 758.16	*F*(000) = 395
Triclinic, *P*1	*D*_x_ = 1.571 Mg m^−^^3^
Hall symbol: -P 1	Mo *K*α radiation, λ = 0.71073 Å
*a* = 6.7353 (3) Å	Cell parameters from 3230 reflections
*b* = 8.0757 (4) Å	θ = 3.0–28.6°
*c* = 15.0170 (6) Å	µ = 0.77 mm^−^^1^
α = 79.450 (4)°	*T* = 100 K
β = 86.320 (4)°	Block, blue
γ = 89.828 (4)°	0.20 × 0.18 × 0.18 mm
*V* = 801.31 (6) Å^3^	

### Data collection

**Table:**

Oxford Diffraction KM-4-CCD diffractometer	3758 independent reflections
Radiation source: fine-focus sealed tube	3230 reflections with *I* > 2σ(*I*)
graphite	*R*_int_ = 0.015
ω scans	θ_max_ = 28.6°, θ_min_ = 3.0°
Absorption correction: analytical (*CrysAlis RED*; Oxford Diffraction, 2010)	*h* = −8→8
*T*_min_ = 0.845, *T*_max_ = 0.910	*k* = −10→10
7090 measured reflections	*l* = −18→20

### Refinement

**Table:**

Refinement on *F*^2^	Primary atom site location: structure-invariant direct methods
Least-squares matrix: full	Secondary atom site location: difference Fourier map
*R*[*F*^2^ > 2σ(*F*^2^)] = 0.030	Hydrogen site location: inferred from neighbouring sites
*wR*(*F*^2^) = 0.082	H-atom parameters constrained
*S* = 1.09	*w* = 1/[σ^2^(*F*_o_^2^) + (0.0454*P*)^2^ + 0.2055*P*] where *P* = (*F*_o_^2^ + 2*F*_c_^2^)/3
3758 reflections	(Δ/σ)_max_ < 0.001
224 parameters	Δρ_max_ = 0.55 e Å^−^^3^
0 restraints	Δρ_min_ = −0.21 e Å^−^^3^

### Special details

**Table:**

Geometry. All e.s.d.'s (except the e.s.d. in the dihedral angle between two l.s. planes) are estimated using the full covariance matrix. The cell e.s.d.'s are taken into account individually in the estimation of e.s.d.'s in distances, angles and torsion angles; correlations between e.s.d.'s in cell parameters are only used when they are defined by crystal symmetry. An approximate (isotropic) treatment of cell e.s.d.'s is used for estimating e.s.d.'s involving l.s. planes.
Refinement. Refinement of *F*^2^ against ALL reflections. The weighted *R*-factor *wR* and goodness of fit *S* are based on *F*^2^, conventional *R*-factors *R* are based on *F*, with *F* set to zero for negative *F*^2^. The threshold expression of *F*^2^ > σ(*F*^2^) is used only for calculating *R*-factors(gt) *etc*. and is not relevant to the choice of reflections for refinement. *R*-factors based on *F*^2^ are statistically about twice as large as those based on *F*, and *R*- factors based on ALL data will be even larger. The X-ray data were collected at 100 K using a KM4-CCD diffractometer and graphite-monochromated MoKalpha radiation generated from Oxford Diffraction X-ray tube operated at 50 kV and 25 mA. The obtained images were indexed, integrated, and scaled using the Oxford Diffraction data reduction package. The structure was solved by direct methods using *SHELXS97* and refined by the full?matrix least-squares method on all F2 data. The data were corrected for absorption [CrysAlis], min/max absorption coefficients for 1 are (0.845/0.910).

### Fractional atomic coordinates and isotropic or equivalent isotropic displacement parameters (Å^2^)

**Table:**

	*x*	*y*	*z*	*U*_iso_*/*U*_eq_	
Cu1	0.0000	1.0000	0.5000	0.01475 (9)	
O1	−0.13388 (16)	0.97838 (14)	0.39113 (7)	0.0154 (2)	
O2	−0.10153 (16)	0.85683 (14)	0.26828 (7)	0.0142 (2)	
O3	0.32384 (16)	0.80385 (14)	0.16138 (7)	0.0153 (2)	
O4	0.16155 (16)	0.56417 (14)	0.22248 (7)	0.0149 (2)	
N1	0.20923 (19)	0.86576 (16)	0.44718 (8)	0.0128 (3)	
N2	0.46299 (19)	0.67808 (16)	0.35325 (9)	0.0139 (3)	
C1	−0.0421 (2)	0.89169 (18)	0.33887 (10)	0.0116 (3)	
C2	0.1593 (2)	0.82665 (18)	0.36827 (10)	0.0115 (3)	
C3	0.2884 (2)	0.73410 (18)	0.32044 (10)	0.0119 (3)	
C4	0.2508 (2)	0.69782 (19)	0.22677 (10)	0.0127 (3)	
C5	0.5068 (2)	0.7161 (2)	0.43260 (10)	0.0155 (3)	
H5	0.6278	0.6762	0.4577	0.019*	
C6	0.3820 (2)	0.8123 (2)	0.48003 (10)	0.0150 (3)	
H6	0.4197	0.8396	0.5355	0.018*	
O1W	−0.14082 (18)	0.72777 (16)	0.57622 (8)	0.0229 (3)	
H1W	−0.2300	0.7321	0.6166	0.034*	
H2W	−0.0443	0.6716	0.5995	0.034*	
N11	0.21766 (18)	0.46151 (16)	0.05780 (8)	0.0118 (2)	
H11	0.2053	0.4960	0.1047	0.014*	
C11	0.2788 (2)	0.56690 (19)	−0.02005 (10)	0.0123 (3)	
C12	0.2921 (2)	0.5011 (2)	−0.10119 (10)	0.0151 (3)	
H12A	0.3349	0.5709	−0.1571	0.018*	
C13	0.2428 (2)	0.3360 (2)	−0.09838 (11)	0.0179 (3)	
H13	0.2489	0.2921	−0.1530	0.021*	
C14	0.1833 (2)	0.2305 (2)	−0.01580 (11)	0.0173 (3)	
H14	0.1516	0.1154	−0.0143	0.021*	
C15	0.1715 (2)	0.29512 (19)	0.06241 (11)	0.0143 (3)	
C16	0.1078 (2)	0.1984 (2)	0.15434 (11)	0.0175 (3)	
H16A	0.2040	0.2170	0.1978	0.026*	
H16B	0.1013	0.0780	0.1520	0.026*	
H16C	−0.0237	0.2365	0.1735	0.026*	
N12	0.32229 (19)	0.72626 (16)	−0.01707 (9)	0.0145 (3)	
H12B	0.3187	0.7575	0.0309	0.017*	
H12C	0.3504	0.7944	−0.0665	0.017*	
O5W	0.48141 (18)	0.24042 (15)	0.31316 (8)	0.0221 (3)	
H5W	0.5811	0.2966	0.3151	0.033*	
H6W	0.5090	0.1774	0.2771	0.033*	
O6W	0.80815 (17)	0.46006 (14)	0.32461 (8)	0.0193 (2)	
H7W	0.7333	0.5356	0.3298	0.029*	
H8W	0.9046	0.5041	0.2935	0.029*	
O7W	0.59226 (16)	1.03986 (14)	0.18630 (7)	0.0168 (2)	
H9W	0.6793	0.9904	0.2109	0.025*	
H10W	0.5095	0.9622	0.1811	0.025*	

### Atomic displacement parameters (Å^2^)

**Table:**

	*U*^11^	*U*^22^	*U*^33^	*U*^12^	*U*^13^	*U*^23^
Cu1	0.01330 (14)	0.02161 (16)	0.01211 (14)	0.00441 (10)	−0.00247 (10)	−0.00986 (10)
O1	0.0136 (5)	0.0207 (6)	0.0143 (5)	0.0036 (4)	−0.0020 (4)	−0.0092 (4)
O2	0.0148 (5)	0.0174 (5)	0.0123 (5)	0.0007 (4)	−0.0031 (4)	−0.0066 (4)
O3	0.0180 (5)	0.0170 (5)	0.0116 (5)	−0.0023 (4)	0.0010 (4)	−0.0051 (4)
O4	0.0158 (5)	0.0153 (5)	0.0150 (5)	−0.0024 (4)	0.0004 (4)	−0.0070 (4)
N1	0.0137 (6)	0.0145 (6)	0.0108 (6)	−0.0004 (5)	−0.0001 (5)	−0.0041 (5)
N2	0.0119 (6)	0.0156 (6)	0.0147 (6)	−0.0005 (5)	−0.0002 (5)	−0.0042 (5)
C1	0.0114 (7)	0.0117 (7)	0.0118 (7)	−0.0008 (5)	0.0000 (5)	−0.0023 (5)
C2	0.0123 (7)	0.0128 (7)	0.0098 (6)	−0.0018 (6)	−0.0007 (5)	−0.0032 (5)
C3	0.0121 (7)	0.0119 (7)	0.0118 (7)	−0.0028 (5)	0.0004 (5)	−0.0028 (5)
C4	0.0090 (6)	0.0169 (7)	0.0138 (7)	0.0031 (6)	−0.0004 (5)	−0.0073 (6)
C5	0.0129 (7)	0.0177 (7)	0.0159 (7)	−0.0008 (6)	−0.0026 (6)	−0.0030 (6)
C6	0.0152 (7)	0.0183 (8)	0.0124 (7)	−0.0014 (6)	−0.0032 (6)	−0.0040 (6)
O1W	0.0195 (6)	0.0287 (7)	0.0193 (6)	−0.0008 (5)	−0.0010 (5)	−0.0013 (5)
N11	0.0120 (6)	0.0141 (6)	0.0105 (6)	−0.0002 (5)	−0.0007 (5)	−0.0054 (5)
C11	0.0080 (6)	0.0157 (7)	0.0140 (7)	0.0014 (5)	−0.0022 (5)	−0.0044 (6)
C12	0.0130 (7)	0.0216 (8)	0.0116 (7)	0.0028 (6)	−0.0008 (5)	−0.0049 (6)
C13	0.0145 (7)	0.0238 (8)	0.0186 (8)	0.0045 (6)	−0.0039 (6)	−0.0116 (6)
C14	0.0149 (7)	0.0145 (7)	0.0248 (8)	0.0008 (6)	−0.0029 (6)	−0.0090 (6)
C15	0.0090 (6)	0.0146 (7)	0.0197 (7)	0.0010 (5)	−0.0020 (6)	−0.0040 (6)
C16	0.0169 (7)	0.0153 (7)	0.0196 (8)	−0.0002 (6)	−0.0004 (6)	−0.0010 (6)
N12	0.0174 (6)	0.0156 (6)	0.0108 (6)	−0.0014 (5)	−0.0007 (5)	−0.0036 (5)
O5W	0.0189 (6)	0.0273 (6)	0.0227 (6)	−0.0005 (5)	−0.0024 (5)	−0.0110 (5)
O6W	0.0181 (6)	0.0179 (6)	0.0211 (6)	0.0005 (5)	0.0031 (5)	−0.0031 (5)
O7W	0.0151 (5)	0.0160 (5)	0.0196 (6)	−0.0008 (4)	−0.0055 (4)	−0.0024 (4)

### Geometric parameters (Å, °)

**Table:**

Cu1—O1^i^	1.9522 (10)	N11—C11	1.3553 (19)
Cu1—O1	1.9522 (10)	N11—C15	1.3685 (19)
Cu1—N1^i^	1.9881 (13)	N11—H11	0.8021
Cu1—N1	1.9882 (13)	C11—N12	1.3301 (19)
Cu1—O1W	2.4484 (13)	C11—C12	1.413 (2)
Cu1—O1W	2.4483 (12)	C12—C13	1.367 (2)
Cu1—O1W^i^	2.4483 (12)	C12—H12A	0.9500
O1—C1	1.2745 (18)	C13—C14	1.406 (2)
O2—C1	1.2360 (17)	C13—H13	0.9500
O3—C4	1.2540 (19)	C14—C15	1.367 (2)
O4—C4	1.2508 (18)	C14—H14	0.9500
N1—C6	1.333 (2)	C15—C16	1.494 (2)
N1—C2	1.3438 (18)	C16—H16A	0.9800
N2—C5	1.3347 (19)	C16—H16B	0.9800
N2—C3	1.349 (2)	C16—H16C	0.9800
C1—C2	1.516 (2)	N12—H12B	0.8045
C2—C3	1.394 (2)	N12—H12C	0.8505
C3—C4	1.525 (2)	O5W—H5W	0.8163
C5—C6	1.392 (2)	O5W—H6W	0.8210
C5—H5	0.9500	O6W—H7W	0.8018
C6—H6	0.9500	O6W—H8W	0.8180
O1W—H1W	0.8314	O7W—H9W	0.7824
O1W—H2W	0.8488	O7W—H10W	0.8577
			
O1^i^—Cu1—O1	179.999 (1)	N1—C6—C5	119.66 (14)
O1^i^—Cu1—N1^i^	83.11 (5)	N1—C6—H6	120.2
O1—Cu1—N1^i^	96.89 (5)	C5—C6—H6	120.2
O1^i^—Cu1—N1	96.89 (5)	H1W—O1W—H2W	109.0
O1—Cu1—N1	83.11 (5)	C11—N11—C15	123.79 (13)
N1^i^—Cu1—N1	180.000 (2)	C11—N11—H11	120.1
O1^i^—Cu1—O1W	90.35 (4)	C15—N11—H11	116.1
O1—Cu1—O1W	89.65 (4)	N12—C11—N11	119.09 (13)
N1^i^—Cu1—O1W	94.44 (5)	N12—C11—C12	123.08 (14)
N1—Cu1—O1W	85.56 (5)	N11—C11—C12	117.82 (13)
O1^i^—Cu1—O1W^i^	89.65 (4)	C13—C12—C11	119.33 (14)
O1—Cu1—O1W^i^	90.35 (4)	C13—C12—H12A	120.3
N1^i^—Cu1—O1W^i^	85.56 (5)	C11—C12—H12A	120.3
N1—Cu1—O1W^i^	94.44 (5)	C12—C13—C14	120.91 (15)
O1W—Cu1—O1W^i^	180.0	C12—C13—H13	119.5
C1—O1—Cu1	115.34 (9)	C14—C13—H13	119.5
C6—N1—C2	119.22 (13)	C15—C14—C13	119.33 (14)
C6—N1—Cu1	128.82 (10)	C15—C14—H14	120.3
C2—N1—Cu1	111.95 (10)	C13—C14—H14	120.3
C5—N2—C3	117.26 (13)	C14—C15—N11	118.80 (14)
O2—C1—O1	126.48 (14)	C14—C15—C16	125.00 (14)
O2—C1—C2	118.29 (13)	N11—C15—C16	116.19 (13)
O1—C1—C2	115.23 (12)	C15—C16—H16A	109.5
N1—C2—C3	120.38 (14)	C15—C16—H16B	109.5
N1—C2—C1	114.31 (13)	H16A—C16—H16B	109.5
C3—C2—C1	125.31 (13)	C15—C16—H16C	109.5
N2—C3—C2	121.00 (13)	H16A—C16—H16C	109.5
N2—C3—C4	115.44 (13)	H16B—C16—H16C	109.5
C2—C3—C4	123.47 (13)	C11—N12—H12B	120.0
O4—C4—O3	126.82 (14)	C11—N12—H12C	119.0
O4—C4—C3	118.06 (13)	H12B—N12—H12C	121.0
O3—C4—C3	115.00 (13)	H5W—O5W—H6W	106.8
N2—C5—C6	122.45 (14)	H7W—O6W—H8W	105.5
N2—C5—H5	118.8	H9W—O7W—H10W	103.7
C6—C5—H5	118.8		
			
N1^i^—Cu1—O1—C1	−178.18 (10)	N1—C2—C3—C4	−174.52 (13)
N1—Cu1—O1—C1	1.82 (10)	C1—C2—C3—C4	5.3 (2)
O1^i^—Cu1—N1—C6	0.11 (14)	N2—C3—C4—O4	91.19 (16)
O1—Cu1—N1—C6	−179.90 (14)	C2—C3—C4—O4	−92.28 (18)
O1^i^—Cu1—N1—C2	179.37 (10)	N2—C3—C4—O3	−85.16 (17)
O1—Cu1—N1—C2	−0.63 (10)	C2—C3—C4—O3	91.36 (17)
Cu1—O1—C1—O2	177.40 (12)	C3—N2—C5—C6	−1.2 (2)
Cu1—O1—C1—C2	−2.51 (16)	C2—N1—C6—C5	−0.4 (2)
C6—N1—C2—C3	−1.3 (2)	Cu1—N1—C6—C5	178.80 (11)
Cu1—N1—C2—C3	179.36 (11)	N2—C5—C6—N1	1.8 (2)
C6—N1—C2—C1	178.89 (13)	C15—N11—C11—N12	−179.23 (13)
Cu1—N1—C2—C1	−0.45 (15)	C15—N11—C11—C12	1.1 (2)
O2—C1—C2—N1	−177.94 (13)	N12—C11—C12—C13	−179.40 (15)
O1—C1—C2—N1	1.98 (19)	N11—C11—C12—C13	0.2 (2)
O2—C1—C2—C3	2.3 (2)	C11—C12—C13—C14	−1.3 (2)
O1—C1—C2—C3	−177.83 (13)	C12—C13—C14—C15	1.1 (2)
C5—N2—C3—C2	−0.5 (2)	C13—C14—C15—N11	0.2 (2)
C5—N2—C3—C4	176.09 (13)	C13—C14—C15—C16	179.08 (14)
N1—C2—C3—N2	1.8 (2)	C11—N11—C15—C14	−1.3 (2)
C1—C2—C3—N2	−178.38 (13)	C11—N11—C15—C16	179.68 (13)

Symmetry codes: (i) −*x*, −*y*+2, −*z*+1.

### Hydrogen-bond geometry (Å, °)

**Table:**

*D*—H···*A*	*D*—H	H···*A*	*D*···*A*	*D*—H···*A*
O1W—H1W···O5W^ii^	0.83	1.97	2.7841 (17)	166
O1W—H2W···O6W^iii^	0.85	2.18	3.0199 (17)	172
O5W—H5W···O6W	0.82	2.05	2.8640 (17)	173
O5W—H6W···O7W^iv^	0.82	1.96	2.7839 (16)	175
O6W—H7W···N2	0.80	2.19	2.9688 (17)	162
O6W—H8W···O4^v^	0.82	1.99	2.7921 (16)	168
O7W—H9W···O2^v^	0.78	1.97	2.7559 (15)	177
O7W—H10W···O3	0.86	1.87	2.7221 (16)	176
N11—H11···O4	0.80	1.95	2.7522 (16)	175
N12—H12B···O3	0.80	2.06	2.8623 (17)	172
N12—H12C···O7W^vi^	0.85	2.05	2.9014 (17)	178
C5—H5···O1W^v^	0.95	2.53	3.3206 (19)	141
C6—H6···O5W^iii^	0.95	2.38	3.2485 (19)	151
C13—H13···O2^vii^	0.95	2.53	3.4081 (19)	153
C16—H16B···O2^iv^	0.98	2.58	3.2419 (19)	125

Symmetry codes: (ii) −*x*, −*y*+1, −*z*+1; (iii) −*x*+1, −*y*+1, −*z*+1; (iv) *x*, *y*−1, *z*; (v) *x*+1, *y*, *z*; (vi) −*x*+1, −*y*+2, −*z*; (vii) −*x*, −*y*+1, −*z*.
